# Reducing Antibiotic Dependence in Poultry: The Potential of Phytochemicals as Antibiotic Alternatives Against Bacterial Foodborne Pathogens

**DOI:** 10.3390/tropicalmed11060153

**Published:** 2026-06-04

**Authors:** Rithu Chandran, Thomas Denagamage, Daniel M. Czyz, Subhashinie Kariyawasam, Abraham Joseph Pellissery

**Affiliations:** 1 Department of Comparative, Diagnostic and Population Medicine, College of Veterinary Medicine, University of Florida, Gainesville, FL 32608, USA; rithuchandran@ufl.edu (R.C.); skariyawasam@ufl.edu (S.K.); 2 Department of Large Animal Clinical Sciences, College of Veterinary Medicine, University of Florida, Gainesville, FL 32608, USA; tdenagamage@ufl.edu; 3 Microbiology and Cell Science Department, College of Agricultural and Life Sciences, University of Florida, Gainesville, FL 32611, USA; dczyz@ufl.edu; 4 Emerging Pathogens Institute, University of Florida, Gainesville, FL 32610, USA; 5 Bronson Animal Disease Diagnostic Laboratory, Kissimmee, FL 34741, USA

**Keywords:** phytochemicals, antimicrobial resistance, poultry, zoonotic pathogens, foodborne bacteria, antibiotic alternatives

## Abstract

Antimicrobial resistance (AMR) is one of the most serious threats to global public health, driven in part by extensive antibiotic use in food-producing animals. The poultry industry, a major contributor to the global animal protein supply, has depended on antibiotics for growth promotion and disease control, thereby contributing to the emergence and dissemination of AMR zoonotic bacteria. This review synthesizes current evidence on the potential of phytochemicals (PCs), plant-derived bioactive compounds, as sustainable non-antibiotic alternatives for controlling bacterial foodborne pathogens in poultry. Relevant literature including in vitro and in vivo studies assessing PCs against major poultry-associated zoonotic bacteria, including *Salmonella enterica*, *Campylobacter* spp., *Clostridium perfringens*, *Listeria monocytogenes*, and pathogenic *Escherichia coli*, is examined. Evidence indicates that PCs exert antimicrobial and anti-virulence effects through mechanisms like bacterial membrane disruption, inhibition of quorum sensing and virulence gene expression, modulation of gut microbiota, and enhancement of host immune responses. In vivo studies demonstrate reductions in pathogen colonization and improvements in gut health and performance metrics in poultry. Despite these promising findings, challenges remain in bioavailability, dose optimization, standardization, and regulatory approval. Overall, PCs represent a promising component of integrated antimicrobial stewardship strategies in poultry production, with significant implications for mitigating zoonotic AMR transmission.

## 1. Introduction

The convergence of antimicrobial resistance (AMR) and foodborne infections constitutes a significant threat to global health security in the 21st century. This is no longer seen as merely a clinical complication but as a macroeconomic challenge that threatens a century of medical progress [1]. AMR-related mortality has reached levels comparable to or exceeding other major global health crises. According to a study that spanned 204 countries, it was estimated that, in 2021, about 1.14 million deaths were caused directly by AMR, and in approximately 4.71 million deaths, AMR infections were a complicating factor. With long-term modelling, it is projected that, if the situation remains unchanged, AMR could lead to more than 39 million deaths between 2025 and 2030 [2]. The Global Antibiotic Resistance Surveillance Report 2025 confirmed that in 2023, one in six laboratory-confirmed bacterial infections worldwide were resistant to antibiotic treatment, based on over 23 million infections reported across 104 countries [3]. Furthermore, resistance levels increased in more than 40% of the pathogen–antibiotic combinations under surveillance between 2018 and 2023, with average annual increases of 5–15%. Foodborne pathogens such as *Salmonella* and *Shigella* continue to be major contributors to the burden, causing widespread gastrointestinal disease. Invasive *Salmonella* infections, for example, may cause case fatality rates up to 15–20% and the increasing resistance is linked to antibiotic use in animal production [3]. Zoonotic foodborne infections facilitate the transfer of AMR bacteria from animal-derived foods to humans [4]. The industrialization of livestock production by intensive farming techniques has contributed to the emergence and spread of drug-resistant zoonotic pathogens. Within this context, the poultry industry occupies a critical position in the One Health interface, linking animal production, environment contamination and human health. The poultry industry has historically relied on antibiotics as growth promoters at subtherapeutic doses apart from their prophylactic and therapeutic uses in intensive production systems [5]. Antibiotic growth promoters (AGPs) have been associated with increased metabolic efficiency and control of subclinical diseases, thereby increasing production [6]. In the early decades of AGP use, under less stringent biosecurity, growth promoters likely produced larger performance gains. But today, many producers achieve high productivity even without AGPs, using improved hygiene, nutrition, and management [7]. Still, AGPs remain attractive in many settings for their prophylactic effects, continuously suppressing low-grade infections (e.g., necrotic enteritis or other enteric bacterial challenges) and thus maximizing production efficiency in high-density flocks [8].

The flip side of the long-term antibiotic supplementation was the strong selective pressure it created on microbial populations, leading to the emergence of AMR in poultry-associated bacteria. Overuse of AGPs has accelerated the development of resistant strains in the poultry gut and farm environment. As a result, many common bacterial pathogens and commensals in poultry, including *Escherichia coli*, *Salmonella* spp., *Campylobacter*, *Staphylococcus aureus*, and *Enterococcus*, developed high levels of resistance due to the widespread and often unregulated use of antibiotics in feed [9]. One example is vancomycin-resistant *Enterococcus faecium* (VRE): the use of the glycopeptide antibiotic avoparcin as a growth promoter in European poultry and pig farms created a large reservoir of *E. faecium* carrying the *vanA* gene, the same resistance determinant that confers vancomycin resistance in human infections [10]. Studies in the 1990s found that flocks raised without AGPs had no VRE, whereas VRE were isolated from most conventional flocks that had long-term exposure to avoparcin. In Denmark, prior to banning avoparcin, 24,000 kg of this antibiotic were used annually in animal feed, orders of magnitude more than the ~24 kg of vancomycin used in human medicine, demonstrating the intense selective pressure for resistance in livestock [11]. Similar trends have been documented with antimicrobial agents: tetracyclines, beta-lactams, macrolides, and even last-resort antibiotics like colistin were historically used as growth promoters in some countries, which led to the rise of resistant genes in poultry bacteria [12]. These resistant organisms often exist as commensals in birds, allowing them to persist and spread silently in poultry facilities. However, they pose a latent risk, as the barn environment becomes a reservoir of AMR genes that can potentially transfer to pathogenic bacteria [13]. These concerns have intensified the search for sustainable alternative compounds that can reduce pathogen burden while minimizing the selective pressure driving AMR in poultry production systems.

This review summarizes the current understanding of AMR dynamics in poultry production and discusses biologically active natural compounds found in plants called phytochemicals (PCs) [14], as antibiotic-sparing interventions in poultry. Specifically, the review entails phytochemical interventions that can reduce pathogen carriage, virulence or post-harvest persistence in major poultry-associated zoonotic bacteria. Unlike conventional antibiotics that typically target a single pathway, PCs exhibit variable mechanisms of action; some act on specific targets, while others affect multiple processes, including membrane disruption, efflux pump inhibition, and quorum sensing suppression, thereby complicating resistance development [15,16,17,18]. The scope includes an analysis of effectiveness against five major foodborne pathogens and the integration of these compounds into the One Health framework. By synthesizing current data, this review provides a roadmap for precision nutrition as a core component of antimicrobial stewardship for the 2025–2030 window.

## 2. AMR in Poultry: Drivers, Dynamics, and Zoonotic Risk

The global poultry industry has undergone a radical transformation over the last decades, shifting from subsistence-based agricultural practices to highly integrated, intensive production systems designed to meet the growing demand for animal protein [19]. This intensification included the routine application of antimicrobial agents, a practice that has inadvertently established poultry production as a primary epicenter for the emergence and dissemination of AMR [20,21]. The poultry sector’s historical reliance on antimicrobials for therapy and growth promotion has faced increasing scrutiny due to concerns about antibiotic residues and MDROs in the food chain [22,23]. The silent pandemic of AMR now represents one of the most significant threats to global public health, with 2019 estimates indicating that bacterial AMR was associated with approximately 4.95 million deaths, of which 1.27 million were directly attributable to bacterial AMR [24]. Within this framework, the poultry sector functions as a biological refinery where constant selective pressure, high animal density, and complex environmental interfaces facilitate the evolution of MDROs [25]. These resistant organisms and their genetic determinants are subsequently disseminated through the food chain, environmental vectors, and direct occupational exposure, challenging the efficacy of critically important human antibiotics like fluoroquinolones and third generation cephalosporins [20,25].

### 2.1. Patterns of Antibiotic Use in Poultry Production

The patterns of antimicrobial use in poultry are diverse, reflecting a complex interplay of economic incentives, farming practices, veterinary requirements, and varying regulatory frameworks across geographic regions [20]. Antibiotics in this sector are deployed across a spectrum of indications, including growth promotion, disease prevention and the treatment of clinical infections. The indiscriminate or inappropriate application of these compounds, particularly at sub-therapeutic levels for extended durations, provides the ideal evolutionary environment for the selection of resistance genes within the commensal and pathogenic microbiota of the poultry gut [25].

#### 2.1.1. Growth Promotion

The historical trajectory of AGPs began in the mid-1940s when researchers identified that the addition of low doses of antibiotics to poultry feed resulted in significantly improved growth rates and feed efficiency. By 1951, the U.S. Food and Drug Administration (FDA) had formally approved the use of these agents as animal feed additives without a prescription, a decision that catalyzed their worldwide adoption [5,26]. AGPs are typically administered at sub-therapeutic concentrations, significantly lower than those required for clinical treatment, to modify the intestinal microbiota and enhance the metabolic efficiency of the host [27]. Recent meta-analyses suggest that the marginal benefit of AGPs may be waning in modern production systems. A comprehensive review of performance indicators showed an overall improvement in the feed conversion ratio (FCR) of only 2.8% due to AGP supplementation, with significant variation attributed to breed, dosage, and geographical location [28]. Furthermore, studies conducted in hygienic environments have demonstrated that the growth-promoting effect is negligible when compared to birds raised under suboptimal biosecurity conditions, where the antibiotics function primarily by counteracting the high microbial load of a contaminated environment [29,30].

#### 2.1.2. Disease Prevention and Therapy

In the context of intensive poultry farming, antibiotics are essential tools for maintaining animal welfare and managing the high risk of infectious disease outbreaks [31]. However, the distinction between therapy and prophylaxis is often blurred in practice. Prophylaxis involves the administration of antimicrobials to healthy animals at risk of infection, typically during high-stress periods such as brooding, transportation, or after vaccination. Metaphylaxis refers to the treatment of an entire flock after a portion has been diagnosed with a disease, which is necessary in high-density housing where pathogens spread rapidly [32]. The prevalence of prophylactic and metaphylactic use is especially common in regions with poor biosecurity. In Nepal, a cross-sectional study of layer farms revealed that while 50.6% of antibiotic use was for treatment, 32.7% was strictly for prophylaxis, often involving fluoroquinolones and macrolides [33]. In Burkina Faso, self-medication is practiced by 74.6% of farmers, with growth promoters utilized in over 93% of operations [34]. Such uncontrolled use is often driven by a lack of access to diagnostic laboratories; for example, in Nepal, only 39.1% of decisions regarding antibiotic usage are based on laboratory reports, with the remainder relying on professional experience, necropsy findings, or telemedicine [33]. Even in developed countries like U.S., before the implementation of recent regulatory changes [35], medically important antibiotics were being sold over the counter and used by producers without veterinary oversight, enabling self-directed antimicrobial use [36].

In developing countries, antibiotics are frequently used for non-therapeutic purposes in poultry production. For example, in India, it is estimated that 70% of the total antibiotics used in poultry are for growth promotion and disease prevention, with only 30% dedicated to actual therapeutic intervention [12]. The misuse of these agents is compounded by environmental mismanagement, where 50% of farms dispose of biological waste in regular trash and 42% discard expired antibiotics directly into the surroundings, facilitating a secondary cycle of resistance selection in soil and water ecosystems [37].

#### 2.1.3. Regulatory Shifts

The global regulatory response to AMR in poultry has been characterized by a tiered approach, with the EU setting the most stringent precedents. Sweden pioneered the prohibition of AGPs in animal feed in 1986, followed by a total EU-wide ban on 25 different AGPs in 2006 [38]. Recent EU mandates, specifically Regulation (EU) 2019/6 and Regulation (EU) 2019/4, have further restricted prophylactic use and reinforced the accountability of veterinarians, who must now justify every antimicrobial prescription based on diagnostic evidence [38,39]. These efforts led to the Farm to Fork Strategy (F2F), which aims for a 50% reduction in antimicrobial sales by 2030 [40]. In the United States, regulatory shifts have been driven by both legislative action and consumer demand. The FDA banned the therapeutic use of fluoroquinolones (specifically enrofloxacin) in poultry in 2005, citing the rise of fluoroquinolone-resistant *Campylobacter* in humans [41,42]. The implementation of Veterinary Feed Directives (VFD) in 2017 effectively ended the use of medically important antibiotics for growth promotion and required veterinary oversight for water-based and feed-based treatments [41]. A concurrent rise in the “Raised Without Antibiotics” (RWA) market segment has pressured producers to adopt non-antibiotic alternatives, although critics note that the lack of robust, federally integrated data collection systems in the U.S. complicates the evaluation of these policies [41,43].

Low and middle-income countries (LMICs) face significant challenges in regulating antimicrobial use [44]. While countries like Mexico and South Korea have moved toward alignment with EU standards, many LMICs still allow the over-the-counter sale of antibiotics without veterinary oversight [45]. The intensification of livestock production in these regions, combined with limited access to modern biosecurity and vaccines, often necessitates the continued use of antimicrobials to maintain food security. It is estimated that agricultural intensification in LMICs will drive a 67% increase in global antimicrobial usage by 2030, highlighting the urgent need for international harmonized guidelines [46].

### 2.2. Selection and Dissemination of Antimicrobial Resistance Genes

**Figure 1:**
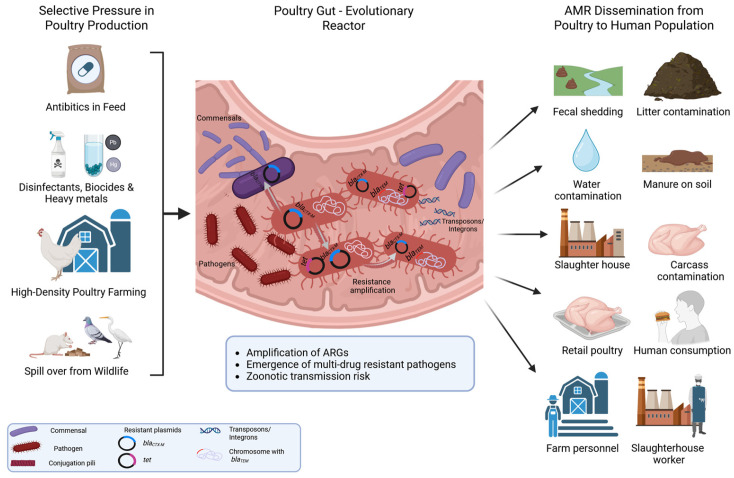
Poultry gut as an evolutionary reactor for antimicrobial resistance (AMR) amplification.

The dissemination of ARGs within poultry production systems is a highly dynamic process facilitated by a sophisticated mobilome. This mobilome encompasses various MGEs like plasmids, integrons and transposons, that allow resistance determinants to move between bacterial chromosomes and extrachromosomal elements, and across diverse bacterial taxa [47,48]. In the high-density environment of intensive poultry farms, the avian gut acts as a bioreactor, where continuous selective pressure from antibiotics accelerates horizontal gene transfer (HGT) between commensal bacteria and zoonotic pathogens [49,50,51]. MGEs facilitate the capture, accumulation, and dissemination of resistance genes [52,53]. Among them, plasmids are the primary vehicles and can carry clinically important ARGs like beta-lactamase genes (*bla*_TEM_, *bla*_SHV-12_, *bla*_CTX-M-15_), as well as last resort resistance genes like *mcr-1* and *bla_NDM-1_* [54,55,56,57,58,59,60,61]. Class I integrons are also commonly detected in poultry-associated *Enterobacteriaceae*, which contribute to dissemination of ARGs [62,63,64]. In MDR *Salmonella*, insertion sequences such as IS26 can further enhance the accumulation and dissemination of ARGs, contributing to resistance against several antibiotic classes like quinolones, aminoglycosides, ampicillin, and chloramphenicol [65]. Transposons further amplify this mobility by transposing these complex resistance clusters between plasmids and the bacterial chromosome, increasing their persistence and spread [65]. The interplay between selective pressures, microbial communities, and MGEs establishes the poultry gut as a dynamic environment for antimicrobial resistance evolution [66]. Within this system, commensal and pathogenic bacteria continuously exchange genetic material through horizontal gene transfer, facilitating the accumulation and dissemination of AMR genes [67,68]. As illustrated in Figure 1, the poultry gut can be conceptualized as an evolutionary resistome reactor, linking agricultural practices to the emergence and downstream dissemination of AMR through environmental, food chain, and occupational pathways.

The poultry intestinal tract functions as a dynamic environment in which selective pressures, microbial communities, and mobile genetic elements (MGEs) interact to drive AMR. External factors, including antibiotics in feed, heavy metals, biocides, intensive farming practices, and spillovers from wildlife, impose selective pressure on the gut microbiota. Within the intestinal lumen, commensal and pathogenic bacteria undergo evolutionary processes such as mutation, horizontal gene transfer (HGT), and gene acquisition, mediated by MGEs including plasmids, integrons, and transposons. These interactions facilitate the amplification and dissemination of antimicrobial resistance genes (ARGs), such as *bla_CTX-M_*, and *tet*. Resistant bacteria and ARGs are subsequently released through multiple pathways, including environmental spread (e.g., fecal shedding, manure application, and water contamination), food chain transmission (e.g., slaughterhouse processing and retail poultry products), and occupational exposure. This integrated system represents a key interface linking antimicrobial use in poultry production to the spread of AMR within the One Health framework (created with BioRender).

### 2.3. Poultry-to-Human Transmission of AMR Bacteria

The transmission of AMR bacteria from poultry to humans follows a complex farm-to-fork trajectory, where resistant organisms and their genetic determinants navigate various biological and environmental barriers to reach the human host [69,70,71]. This zoonotic risk is not merely theoretical; genomic and phylogenetic evidence increasingly confirms the presence of identical high-risk clones in both poultry production chains and human clinical settings, highlighting the interconnected nature of the One Health interface [72,73]. The transition of AMR from the farm environment to humans occurs mainly through three pathways: the consumption of contaminated meat, direct occupational contact, and environmental dissemination via waste products.

#### 2.3.1. Farm-to-Processing Contamination

The colonization of poultry begins at the farm, where fecal shedding leads to high concentrations of resistant pathogens like *Salmonella* and *Campylobacter* in the litter [74,75]. Biofilms formed in the watering systems and on the equipment serve as persistent reservoirs, protecting bacteria from standard sanitation protocols [76,77]. During slaughter, the high processing speed and the mechanical nature of evisceration and defeathering frequently result in cross-contamination of carcasses. In Zimbabwe, while only 3.0% of farm samples were positive for *Salmonella*, the contamination rate jumped to 11% at the slaughterhouse and 20% at the retail market, illustrating the amplification effect of the processing chain [78].

#### 2.3.2. Retail and Consumer Exposure

Retail poultry products are the final vehicle for human exposure. Consumers are exposed through the handling of raw meat, the cross-contamination of kitchen surfaces, or the consumption of undercooked products. Chicken samples collected from retail stores were found to contain MDR *Salmonella* [79], MDR *E. coli* [80] and antibiotic-resistant *Campylobacter* [81]. In Italy, genomic surveillance in 2023 showed that *Campylobacter jejuni* and *Campylobacter coli* isolates from retail broiler carcasses frequently harbored ciprofloxacin resistance linked to *gyrA* mutations (80.1%) and tetracycline resistance attributed to *tet* genes (64.6%) [82].

#### 2.3.3. Occupational and Environmental Pathways

Farm workers and slaughterhouse personnel are at high risk of direct zoonotic transmission [83,84]. Occupational exposure has been associated with the nasal and dermal carriage of livestock-associated methicillin resistant *Staphylococcus aureus* (LA-MRSA) and extended spectrum beta-lactamase (ESBL)-producing *E. coli* [85,86]. Furthermore, the application of untreated poultry manure as fertilizer and the discharge of farm wastewater into local aquatic ecosystems facilitate the transfer of ARGs and AMR bacteria into the broader environment, where they can eventually re-enter the human food chain through contaminated water or crops [87].

## 3. The Poultry Gut Microbiome as a Reservoir and Amplifier of AMR

The poultry gut harbors a plethora of diverse microbes that carry ARGs, which may get amplified and disseminated into the environment and food chain [66,88,89]. The intensive farming system with inadvertent use of antibiotics combined with co-selective pressures such as metals and biocides gives rise to a rich gut resistome [90]. The mobility and composition of the gut resistome depend on the microorganisms and their MGEs [66]. A study in China that analyzed metagenomes of 629 chicken gut samples found genes that confer resistance to tetracyclines, macrolides, aminoglycosides, beta-lactams, and last-resort antibiotics. This study also discovered a linear correlation between the abundance of ARGs and MGEs. These ARGs, which included *tetX*, *mcr*, and *bla*_NDM_, were carried mostly by *Escherichia*, *Enterococcus*, *Staphylococcus*, *Klebsiella*, and *Lactobacillus* [66].

Pathogenic and opportunistic strains of *E. coli*, *Salmonella*, and *Campylobacter*, which cause foodborne illness in humans, exhibit high rates of multidrug resistance, including ESBL production, and resistance to fluoroquinolone, tetracyclines and macrolides [91,92], with overlapping resistance phenotypes seen in isolates from poultry and farm workers or consumers [93]. Detection of last-resort resistance genes such as *mcr* and *bla*_NDM_ in poultry-associated *E. coli* and other Enterobacterales highlights the potential for the poultry gut microbiome to serve as a bridge for clinically critical AMR determinants into human populations [66]. A study that focused on poultry reared under consistent management found that AMR diversity tends to decrease over time while the frequency of specific ARGs rises, indicating selective amplification of particular high-resistance carriers and resistance gene combinations [94].

## 4. Public Health Implications of Poultry-Origin AMR

The public health consequences of poultry-origin AMR are categorized by the loss of therapeutic efficacy in human medicine and the emergence of high-risk zoonotic pathogens. Once established in poultry, antibiotic-resistant bacteria can disseminate beyond the farm, threatening human health. For example, fluoroquinolone antibiotics were used in broiler flocks for disease prophylaxis in the 1990s; subsequently, fluoroquinolone-resistant *Campylobacter* strains became increasingly common in human infections and were epidemiologically linked to poultry consumption. This led regulators to withdraw fluoroquinolone use in poultry to preserve antibiotic efficacy [95]. Resistant *Salmonella* from poultry meat has similarly caused outbreaks of drug-resistant salmonellosis in people [96,97,98]. Even commensal bacteria like *E. coli* from poultry can colonize the human gut through the food chain or direct exposure, carrying over resistance genes that may later transfer to human-adapted pathogens [99,100,101,102]. The public health risk is that infections in humans caused by these zoonotic bacteria (or by human pathogens that acquired resistance genes from animal sources) become more difficult to treat, leading to higher morbidity, mortality, and healthcare costs [103].

### 4.1. Pathogen-Specific Clinical Risks

- *S. enterica*: The most frequently detected MDR pathogen in poultry supply chains is *Salmonella*, particularly serovars like *S*. Typhimurium, *S*. Enteritidis, and *S.* Kentucky ST198. *S.* Kentucky ST198 is a particularly concerning clone that produces ESBL and has been linked to severe infections in humans [104,105,106]. The National Antimicrobial Resistance Monitoring System (NARMS) interim analyses emphasize that rising decreased susceptibility among *S.* Enteritidis is clinically relevant because it may adversely affect fluoroquinolone-treated cases, and NARMS genomic analyses indicate commercial chicken products as a likely source for key strain clusters [107].
- *C. jejuni*/*coli*: *Campylobacter* is the leading cause of bacterial gastroenteritis worldwide, with poultry recognized as the primary reservoir [108,109]. From an AMR perspective, the most consequential public health signal is loss of efficacy of macrolides (erythromycin/azithromycin), which are standard first-line options for severe campylobacteriosis. In Italy, researchers recently detected the *erm*(N) gene, a marker of erythromycin resistance, for the first time in a food-origin isolate. This gene was located on the chromosome of *C. coli* isolated from poultry carcasses at slaughterhouses, representing a significant escalation in resistance to a first-line treatment [82].
- Extraintestinal pathogenic *E. coli* (ExPEC): Poultry products are significant reservoirs for ExPEC, which cause neonatal meningitis, bacteremia, and most human urinary tract infections (UTIs). A comparative study in Canada reported genetic relatedness between *E. coli* from abattoir animals, particularly chickens, and ExPEC from human urinary tract infections, concluding that chickens were the most probable reservoir among animals sampled [110]. Studies using Caco-2 human epithelial cells have shown that 62.8% of poultry-isolated ExPEC can adhere to human intestinal tissues as effectively as known enteric pathogens, suggesting their high potential for establishing extraintestinal infections after intestinal colonization [111].

### 4.2. Genomic Evidence of Zoonotic Linkage

Whole-genome sequencing (WGS) has revolutionized the ability to track AMR transmission. Phylogenetic analysis confirmed identical core single nucleotide polymorphisms (SNPs) across different stages of the broiler supply chain, proving that specific MDR clones move from the farm to the consumer [78]. Similarly, core-genome multilocus sequence typing (cgMLST) showed that 40% of *Campylobacter* isolates formed genetic clusters containing both human clinical and retail food isolates, suggesting potential nationwide outbreak scenarios [82]. However, global studies show varying degrees of compartmentalization; for instance, while ESBL-*E. coli* ST131 and ST10 are common in both Swiss wastewater and clinics, genetic similarity to animal isolates is often restricted by ecological boundaries in high-income regions [112]. However, in India, high-risk clones like ST167 and ST117 are frequently found co-circulating in both poultry and human clinical environments, likely due to more frequent inter-compartmental exchange [70].

These transmission pathways collectively reflect the farm-to-fork dissemination routes illustrated in Figure 1.

## 5. PCs as Non-Antibiotic Interventions in Poultry Production

The increase in public demand for organic meat products and “raised without antibiotics” labels has led to the search for a bioactive alternative that can maintain production yields and health of poultry without using drugs or hormones. PCs encompass non-nutritive secondary plant metabolites [113]. PC feed additives have emerged as a leading candidate, especially as many of them show multifaceted biological effects that target bacterial virulence mechanisms, host immune responses and the gut microbiome, when compared to synthetic antibiotics, which usually have one mechanism of action. They have been used in traditional medicine by ancient civilizations across various cultures, valued for their medicinal properties such as anti-inflammatory and antimicrobial effects [114]. They are generally recognized as safe (GRAS) and are used as feed additives [115]. They can be broadly grouped into carotenoids, phenolics, alkaloids, nitrogen-containing compounds, and organo-sulfur compounds, with carotenoids and phenolics being the most extensively studied [116]. Research efforts to substitute PCs for antibiotics in animal diets have grown as their mechanistic studies in animals show some promise [115].

### 5.1. Mechanisms of Action of PCs Relevant to AMR Mitigation

**Figure 2:**
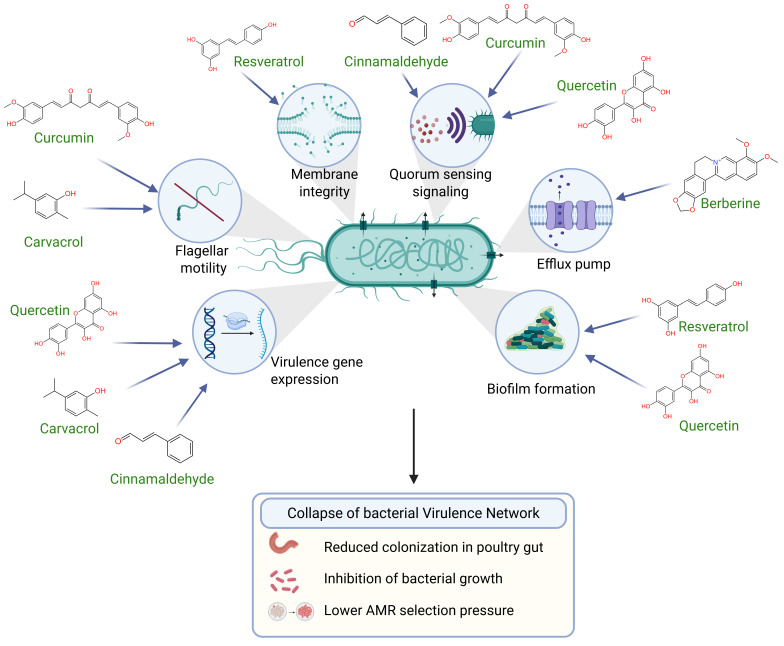
Disruption of bacterial virulence pathways by phytochemicals (PCs).

PCs, which include alkaloids, polyphenols, terpenes, and organosulfur compounds, often have multi-target effects, which makes them different from conventional antibiotics, as illustrated in Figure 2. They target the structural, genetic and cell communication mechanisms of microbial survival.

PCs can modulate bacterial survival and virulence through simultaneous effects on multiple cellular pathways, in contrast to conventional antibiotics that typically target a single site. Key processes affected include membrane integrity, quorum sensing, efflux pump activity, motility, biofilm formation, and virulence gene expression. Representative examples include carvacrol, which can disrupt membrane structure and virulence gene expression; quercetin, which can interfere with quorum sensing signaling, biofilm formation and virulence gene expression; berberine, which inhibits efflux pumps; curcumin, which can reduce motility and quorum sensing activity; resveratrol, which targets biofilm formation and membrane integrity; and cinnamaldehyde, which can modulate virulence gene expression and disrupt quorum sensing. The disruption of these pathways leads to growth inhibition, reducing bacterial colonization and pathogenicity. This multi-target mode of action may also reduce selective pressure for resistance development, supporting the use of PCs as complementary strategies for antimicrobial stewardship in poultry production systems (created with BioRender).

#### 5.1.1. Disruption of Bacterial Cell Membranes

The cell membrane, being a semipermeable barrier, plays an essential role in maintaining the internal chemical environment of the cell and its viability [117]. Unlike the antibiotics that attack cell wall synthesis or specific metabolic enzymes required for cell wall synthesis, membrane-active PCs interact directly with the lipid bilayer or membrane-bound proteins, making resistance development difficult [118].

The amphiphilic or hydrophobic structures of certain PCs allow them to intercalate into the lipid bilayer, which alters the physicochemical properties of the membrane [15,119]. A phenolic compound, gallic acid, is known to cause membrane disruption in Gram-negative bacteria and chelation of Mg^2+^, which further destabilizes the outer membrane [120]. Cannabidiol has been shown to modify the transition temperature, cohesion enthalpy and cooperativity of the lipids, causing disruption of the membrane [119]. Scanning electron microscopy of bacteria treated with PCs has confirmed the shrinkage and disruption of the cell membrane [121,122,123].

Beyond generalized disruption of the lipid matrix, specific PCs target the enzymatic and transport functions located within the membrane. Tomatidine, an alkaloid found in solanaceous plants, serves as a potent inhibitor of membrane-bound ATP synthase in several Gram-positive pathogens, including *Listeria* and *Bacillus* species [124,125]. By blocking the conversion of ADP to ATP, tomatidine effectively starves the bacteria of energy, particularly under conditions where oxidative phosphorylation is the primary energy source [125]. Similarly, sulforaphane, an organosulfur compound, has been shown to cause membrane destruction while simultaneously inhibiting ATP synthase and interfering with DNA and protein synthesis [126]. The inhibition of efflux pumps is a critical mechanism by which PCs mitigate AMR [18]. Efflux pumps are membrane-bound transport proteins that actively expel a wide range of antibiotics from the bacterial cell, thereby reducing their effective concentration at intracellular targets [18,127,128]. PCs such as berberine, conessine, and reserpine have demonstrated potential as efflux pump inhibitors [129,130]. Blocking these transporters enhances the intracellular accumulation of antibiotics, consequently reversing resistance in strains that rely on efflux mechanisms [131].

#### 5.1.2. Inhibition of Quorum Sensing

Quorum sensing (QS) is a sophisticated intercellular communication system that allows bacteria to monitor their population density through production, detection, and response to signaling molecules called autoinducers (AIs) [132,133]. When a threshold concentration of these signals is reached, the bacterial population undergoes a coordinated change in gene expression, activating pathways related to virulence factor production, biofilm maturation, and antibiotic tolerance [134]. Because QS orchestrates pathogenic behaviors without being essential for primary growth, its inhibition offers an attractive anti-virulence strategy that reduces the selective pressure for resistance development [16,133,135,136].

PCs act as quorum sensing inhibitors (QSIs) by targeting various stages of the signaling cascade, including signal biosynthesis, signal perception, and downstream regulatory cascades [136]. In Gram-negative bacteria, the most common QS system involves acyl homoserine lactones (AHLs) produced by LuxI-type enzymes and detected by LuxR-type receptors [137,138]. PCs such as carvacrol have been shown to downregulate the expression of the *cviI* gene (a LuxI homolog) in *Chromobacterium violaceum*, leading to a significant reduction in AHL production and the suppression of QS-dependent outputs like violacein synthesis [139]. In *Pseudomonas aeruginosa*, quercetin and rosmarinic acid interfere with the *lasI/lasR* and *rhlI/rhlR* systems, which coordinate the expression of extracellular toxins and proteases [140,141]. In Gram-positive bacteria, QS is typically mediated by autoinducing peptides (AIPs) through the accessory gene regulator (*agr*) system [142]. Curcumin and resveratrol have been identified as modulators of this system in *S. aureus*, where they downregulate the biosynthesis of AIPs and impair the activation of the *agr* signaling network [16,143,144,145]. Rosemary extracts containing carnosic acid and carnosol also exhibit potent QS inhibitory activity against *S. aureus*, effectively attenuating the expression of alpha-toxin and other virulence factors [146].

The LuxS/AI-2 system facilitates interspecies communication between a wide variety of Gram-positive and Gram-negative bacteria. The LuxS enzyme catalyzes the synthesis of AI-2 from S-ribosylhomocysteine, and this signal is recognized by receptors like LuxP and LsrB [147]. PCs such as carnosol and chlorogenic acid have been demonstrated to inhibit the LuxS enzyme directly, as shown through molecular docking and biochemical assays [148]. Furthermore, compounds like carvacrol cinnamaldehyde and eugenol decrease the expression of the *luxS* gene in uropathogenic *E. coli* (UPEC), resulting in reduced motility and biofilm development [149].

#### 5.1.3. Anti-Biofilm and Anti-Motility Effects

Biofilms are highly organized bacterial communities encased in a self-produced matrix of extracellular polymeric substances (EPS), which provides a protective environment against antibiotic penetration and the host immune response [150]. Biofilm-associated bacteria can be up to 1000 times more resistant to antibiotics than their planktonic counterparts, making biofilm eradication a critical challenge in the treatment of chronic and hospital-acquired infections [151]. PCs offer a multi-stage approach to biofilm control, targeting attachment, matrix stability, and dispersal. PCs such as 7-hydroxycoumarin and indole-3-carbinol have been shown to impair the motility and adhesive potential of *E. coli* and *S. aureus* [152]. These compounds often induce structural changes in extracellular appendages such as pili and fimbriae, preventing the stable attachment required for biofilm initiation [152]. Additionally, phloretin and ginkgolic acid have been reported to control the expression of curli and pili genes, hindering surface colonization [153,154]. The EPS matrix consists of various components, including polysaccharides, proteins, and extracellular DNA (eDNA), which collectively provide structural stability and act as a barrier to antimicrobial agents [150]. PCs can disrupt the synthesis of these components, thereby weakening the biofilm’s integrity [155]. For example, luteolin, a phenolic flavone, reduces the synthesis of polysaccharides and eDNA in *E. coli* and *Enterobacter cloacae* biofilms, resulting in significantly reduced biomass [156]. Quercetin has also been demonstrated to lower the abundance of extracellular polysaccharides in *P. aeruginosa* by interfering with the *rhl* transcriptional regulators [157]. Nanoparticles made of gold, silver, or chitosan can enhance the bioavailability and controlled release of PCs, ensuring that bioactive concentrations are achieved at the site of infection [158]. For example, epigallocatechin gallate–silver nanoparticles have been explored as antimicrobial coatings for medical devices, such as catheters and implants, to prevent the formation of hospital-acquired biofilms [159].

#### 5.1.4. Modulation of Gut Microbiota Composition

The gastrointestinal tract is home to a vast and diverse microbial community that is essential for nutrition, metabolic health, and the development of the host immune system [160,161,162]. PCs and the gut microbiota (GM) engage in a bidirectional interaction: the GM metabolizes complex PCs into smaller, more bioavailable molecules, while the PCs, in turn, act as prebiotics to reshape the community structure [163,164]. This modulation is critical for AMR mitigation because the gut serves as a significant reservoir of ARGs that can be transferred between commensal and pathogenic bacteria [67,68].

Many PCs, particularly large polyphenolic glycosides, are poorly absorbed in the small intestine, with 90–95% reaching the colon intact [165]. In the colon, these compounds are metabolized by microbial enzymes to produce highly bioactive constituents; for instance, isoflavones such as genistein or daidzein are converted into equol metabolites by *Slackia isoflavoniconvertens*, which possess significantly greater biological activity than the precursors [166,167]. PCs promote the growth of beneficial bacteria, such as *Bifidobacterium* and *Lactobacillus*, while inhibiting the proliferation of potential pathobionts like *S. aureus* and members of the *Enterobacteriaceae* family [168,169,170]. Anthocyanins from purple sweet potatoes and black rice have been shown to increase the prevalence of *Lactobacillus-Enterococcus* and *Bifidobacterium* species in both in vitro and in vivo models [171,172]. The composition of the GM is a major determinant of the resistome burden [173]. High dietary intake of fiber and PCs is negatively correlated with the abundance of ARGs in the fecal metagenome [174,175].

#### 5.1.5. Enhancement of Host Immune Responses

The efficacy of PCs in mitigating AMR is not limited to their direct antimicrobial actions; it also involves the potentiation of the host’s own defense mechanisms. PCs act as immunomodulators that can balance inflammatory responses, stimulate adaptive immunity, and induce protective mucosal defense, all of which contribute to the clearance of resistant infections [176,177]. PCs can counteract the suppression of innate immunity often induced by pathogenic microbes. For example, carotenoids have been shown to modulate the expression of immunoglobulin A (IgA), a critical factor in the colonization resistance of mucosal surfaces [178]. By enhancing the production of IgA, PCs can prevent the initial attachment of pathogens and the subsequent development of systemic infections [178,179]. Furthermore, many PCs exhibit antioxidant and anti-inflammatory properties that prevent the cytokine storm associated with sepsis [180,181,182,183]. In models of *S. aureus* skin infection, the lichen-derived saponin metabolite 18-beta-glycyrrhetinic acid has demonstrated significant bactericidal and immunomodulatory activity. This compound reduces the expression of bacterial virulence genes like *hla* (alpha-toxin) and *sbi* (antibody evasion), while simultaneously enhancing the host’s ability to resolve soft tissue infections [184].

### 5.2. Additional Benefits of PCs in Poultry Production

Beyond their antimicrobial properties, PCs offer several additional benefits in poultry production, which includes growth performance enhancement by stimulating digestive enzymes, improving gut morphology, and optimizing nutrient absorption. It also helps in gut health modulation through the promotion of beneficial bacteria and inhibition of pathogens, maintaining a balanced microbiota [185]. Many PCs possess antioxidant properties that neutralize free radicals and reduce oxidative stress, supporting immune function and enhancing product quality [186]. While some PCs have anti-inflammatory effects that alleviate oxidative stress-induced inflammation [187], other PCs help stimulate immune cells and cytokine production which improves disease resistance [188]. Furthermore, they play a role in stress mitigation, improving overall bird welfare and productivity [183,189]. In-feed PC supplementation aids in product quality improvement, including better flavor, texture, and nutritional value of meat and eggs [190,191]. Certain PCs also enhance reproductive performance, such as by increasing egg production, fertility, and hatchability rates [192,193]. Moreover, PCs contribute to AMR mitigation by reducing the need for AGPs, offering a natural, biodegradable, and eco-friendly alternative that aligns with consumer demands for antibiotic-free, organic, and sustainable poultry products [123,194]. However, these beneficial effects are compound-specific and vary depending on the type of PC, dosage, formulation and production conditions.

## 6. Effects of PCs Against Major Poultry-Associated Zoonotic Pathogens

The poultry industry contributes significantly to the demand for high-quality protein but at the same time, it serves as a reservoir for zoonotic pathogens. Plant extracts, essential oils and PCs have been extensively studied in poultry research for growth promotion, and treatment and control of diseases [17,114]. Across different pathogens, PCs exhibit antimicrobial, antibiofilm, anti-motility, and anti-virulence effects. But their efficacy depends on the PC compound, extraction method used, formulation, dosage, delivery method used, pathogen strain, bird age and environmental conditions. Additionally, studies employ different experimental designs, from in vitro to controlled animal experiments, making direct comparisons difficult. While several PCs prove to be efficient in reducing pathogen colonization or virulence, complete replacement of antibiotics with PCs remains a challenge due to the variability in bioavailability and stability of the compound. A summary of major PCs, target pathogens, mechanism of action and key findings discussed in this section are given in Table 1.

### 6.1. S. enterica

Based on CDC data, non-typhoidal *Salmonella* holds the top position among 31 foodborne pathogens in the U.S., when ranked by annual illness-related economic burden, with estimated costs reaching $17.1 billion per year [195]. Consumption of contaminated food accounts for 85% of these infections [196]. *Salmonella* isolates resistant to several antibiotic classes, including tetracyclines, β-lactams, aminoglycosides, sulfonamides, and fluoroquinolones, have been found from poultry and poultry products, according to surveillance data [97].

Several studies on *Salmonella* have targeted the suppression of intestinal colonization, motility and virulence-related invasion pathways, compared to other pathogens. Curcumin, resveratrol, eugenol and trans-cinnmaldehyde have been shown to inhibit growth, biofilm formation, motility and membrane integrity of *Salmonella* [197,198,199,200]. In particular, trans-cinnamaldehyde and eugenol were found to reduce cecal colonization and fecal shedding in broilers without significant disruption of native gut microbiota, indicating potential utility as pre-harvest interventions [200]. Efficacy varies between studies according to concentration used, formulation and administration period. In addition, most of these studies assess short-term reduction in colonization under controlled experimental conditions, while long-term field efficacy and consistency are still not sufficiently characterized.

### 6.2. Campylobacter spp.

*Campylobacter* remains a major foodborne concern linked strongly to poultry, with the CDC estimating ~1.5 million illnesses annually in the U.S. [201]. Unlike *Salmonella,* where systemic invasion and intestinal colonization are major concerns, control of *Campylobacter* primarily focuses on reducing cecal carriage and carcass contamination before slaughter. Antibiotic resistance adds urgency to upstream control, especially because severe human campylobacteriosis may require antimicrobial therapy [202,203,204].

Terpenoids like carvacrol exhibit potent anti-motility and anti-colonization effects against *C. jejuni* in vitro [205]. But its in vivo efficacy is highly dependent on dose, delivery and outcome measured (ceca vs. colon/cloaca). For instance, some dietary carvacrol studies reduced cloacal and colon counts but did not significantly reduce cecal colonization at slaughter age [206], while targeted-release formulations were able to show higher reductions in cecal loads [207]. Post-harvest applications such as PC based carcass washes may also represent practical intervention points for *C. jejuni* [208].

### 6.3. Clostridium perfringens

In poultry production, *C. perfringens* is primarily associated with necrotic enteritis (NE), a disease that gained significance after the ban of AGPs [209,210]. It is also a major human foodborne agent [211]. CDC estimates that nearly 1 million foodborne illnesses per year in the U.S. are attributable to *C. perfringens* [212]. This dual relevance (animal health and foodborne illness) is why PC strategies that reduce intestinal overgrowth and tissue damage in poultry are often framed as both productivity and public health interventions [213,214,215]. Improvements in gut lesion scores, intestinal morphology and inflammatory markers are consistently observed with studies using thymol, carvacrol, tannic acid and essential oil blends in NE challenge models [216,217,218,219,220,221,222,223,224]. In *C. perfringens* studies, many of the positive effects appear to be mediated through combined antimicrobial, anti-inflammatory and barrier supportive mechanisms rather than simple bactericidal effects [221,224]. However, efficacy is still influenced by formulation stability and intestinal delivery as the volatile compounds can lose activity within the gastrointestinal environment. In an NE challenge evaluation of a targeted-release blend combining organic acids with PC actives, performance and gut health outcomes varied by inclusion rate, with some inclusion levels yielding better overall results compared with challenged controls, supporting the concept that delivery format and dose optimization can be as important as the choice of active compounds [223].

### 6.4. Listeria monocytogenes

*Listeria monocytogenes* presents distinct control challenges compared with enteric poultry colonizers because the primary poultry-associated issue is often processing-environment persistence and refrigerated food survival [225] rather than high-level intestinal carriage in broilers [226,227]. Clinically, it remains one of the most severe foodborne infections: the CDC notes that listeriosis is a leading cause of death from foodborne illness in the U.S., with ~172 deaths/year; nearly all cases are hospitalized, and case-fatality is estimated at ~20% [228]. Poultry-related isolate studies further underline the resistance/biofilm context in which PC interventions are considered [229]. In an analysis of *L. monocytogenes* isolated from fresh retail chicken meat, a high proportion of isolates were categorized as multidrug-resistant and most exhibited biofilm-forming ability [230], which complicates control efforts in poultry processing systems.

Cinnamon bark oil and combinations of carvacrol, thymol and cinnamaldehyde have shown antimicrobial and anti-virulence effects in poultry meat models [231]. However, evidence from poultry-meat model systems shows that PC performance can be matrix- and temperature-dependent [231,232]. Recent work continues to expand mechanistic focus toward sub-MIC antibiofilm effects (e.g., EPS reduction and weakened biofilm cohesion) and testing of virulence attenuation endpoints, suggesting a research shift from “kill curves only” toward “persistence and contamination ecology” control targets [233,234].

### 6.5. Pathogenic E. coli and Other Emerging Zoonotic Bacteria

Pathogenic *E. coli* in poultry-relevant zoonotic infections include: (i) classic foodborne pathotypes such as Shiga toxin-producing *E. coli* (STEC) and (ii) ExPEC, where poultry meat and chicken have been investigated as potential reservoirs for lineages linked to human urinary tract and other extraintestinal infections [235]. A comparative genomic/epidemiologic study suggested that chickens may serve as reservoirs for pathogenic *E. coli* that causes human UTIs [110]. Within poultry production, the key animal-health driver is often avian pathogenic *E. coli* (APEC), responsible for colibacillosis and commonly exhibiting extensive AMR, and a broad virulence repertoire, along with zoonotic potential [236].

PC studies against APEC tend to focus on combined antimicrobial and gut health supportive effects. Cinnamon essential oils, resveratrol, combinations of alkaloids and multi-herb formulations have been shown to have inhibitory activity against poultry-derived *E. coli* isolates, including reductions in biofilm, intestinal bacterial load and disease severity [237,238,239]. Further, in vivo studies show that some PC formulations have antimicrobial effects as well as improving intestinal morphology barrier-related parameters, indicating that modulation of host response may be adding to their effectiveness [239,240]. Synergistic approaches are also being explored increasingly; for example, matrine (an alkaloid) combined with berberine hydrochloride showed enhanced antibacterial activity against MDR APEC isolates both in vitro and in vivo [241]. Moreover, the inhibition of multidrug efflux systems by berberine suggests that some PCs may be involved in reverting antibiotic susceptibility in resistance strains [242]. Finally, other emerging zoonotic bacteria associated with poultry systems warrant attention because AMR priority lists increasingly emphasize pathogens beyond the classic enterics (e.g., methicillin-resistant *S. aureus* (MRSA) and VRE). Updated priority-pathogen lists and AMR surveillance continue to motivate screening of PCs for activity against these organisms, particularly for anti-virulence and anti-biofilm activity that matter in both animal and processing environments. For example, trans-cinnamaldehyde, thymol, and carvacrol have been reported to inhibit growth and downregulate virulence-gene transcription in MRSA isolates in vitro, suggesting potential roles in hygiene adjuncts or topical/preharvest strategies when supported by safety and residue assessments [243].

**Table 1:** Summary of in vitro and in vivo antimicrobial and anti-virulence effects of purified phytochemicals against poultry-associated zoonotic pathogens.

Bacterial Species (Strain/Isolate)	Phytochemical Used	Concentration Studied	Model Details	Other Effects	Reference
*Salmonella enterica* serovar Typhimurium ATCC 14028	Gallic acid	MIC 3.5 mg/mL; MBC 4.5 mg/mL.	In vitro planktonic susceptibility assay (Broth microdilution in LB broth)	Tested at sublethal levels for membrane integrity/permeability and antivirulence phenotypes; study explicitly addresses antivirulence and antimicrobial effects	[244]
	Protocatechuic acid	MIC 2.0 mg/mL; MBC 2.0 mg/mL			
	Vanillic acid	MIC 1.5 mg/mL; MBC 2.0 mg/mL			
*S.* Typhimurium ATCC14028	Resveratrol	MIC 250 µg/mL.	In vitro MIC (Broth Microdilution); mechanistic assays	Cell wall/membrane structural damage and metabolomics-linked effects	[199]
*S.* Enteritidis PT8	trans-Cinnamaldehyde	SIC: 0.01% (~0.75 mM)	In vitro transcriptomics at SIC dose	Downregulated genes related to motility, SPI-1 regulation, invasion, transport/outer membrane proteins; upregulated heat shock genes	[245]
	Eugenol	SIC: 0.04% (~2.46 mM)			
*S.* Enteritidis	Trans-Cinnamaldehyde	Low dose: 0.5% High dose: 0.75% (in-feed)	In vivo broiler chicken colonization model (*n* = 75/experiment), inoculated on day 8 with ~8 log_10_ CFU/bird, followed through 10 days; cecal enumeration at days 7 and 10 post-inoculation.	≥3 log_10_ CFU/g reduction in cecal *S.* Enteritidis after 10 days of infection; SIC exposure led to reduced motility/invasion, downregulation of motility/invasion genes	[246]
*S.* Enteritidis	Eugenol	Low dose: 0.75% High dose: 1% (in-feed)		≥3 log_10_ CFU/g reduction in cecal *S.* Enteritidis after 10 days of infection; SIC exposure led to reduced motility/invasion, downregulation of motility/invasion genes; lower body weights vs. controls	
*S.* Enteritidis	Trans-Cinnamaldehyde	0.75% in-feed for 5 days pre-slaughter	In vivo commercial market-age broilers; gavage challenge on day 30; euthanasia day 31.	Reduced colonization and shedding	[200]
*S.* Enteritidis	Eugenol	0.1% in-feed for 5 days pre-slaughter			
*S.* Typhimurium (CVCC541)	Thymol	MIC 375 µg/mL; MBC 750 µg/mL	In vitro broth dilution method	Checkerboard assay; combination of thymol and carvacrol showed additive effect	[216]
*S.* Typhimurium (CVCC541)	Carvacrol	MIC 375 µg/mL; MBC 750 µg/mL			
*Campylobacter jejuni* (S-8, NCTC 81-176)	Carvacrol	SIC: 0.002%	In vitro motility assay Adhesion assay LC-MS/MS proteomics	Reduced motility and adhesion, decreased AI-2 activity, increased acid/bile susceptibility; reduced expression of proteins linked to motility/adhesion/ metabolism/ respiration.	[205]
*C. jejuni*	Carvacrol	120 mg/kg in-feed	In vivo broiler trial: seeder bird model through slaughter age	Reductions in cloacal swab loads and colon counts during early periods; no significant reduction in cecal counts at day 33	[206]
*C. jejuni*	Carvacrol	0.25%, 0.5%, 1%, 2% (suspension)	Ex vivo/food model: chicken skin inoculation	2% wash reduced *C. jejuni* by ~2.4–4 log_10_ CFU/sample; emulsion/nanoemulsion not consistently superior to suspension	[208]
*C. jejuni* (five wild-type isolates)	Caprylic acid	In-feed supplementation at 0.35%, 0.7%, 1.4%, 2.8% for the final 72 h of a 15-day trial	In vivo: day-of-hatch broiler chicks (*n* = 60/trial)	0.7% and 1.4% caprylic acid consistently produced 3–4 log_10_ reductions in cecal counts vs. positive controls	[247]
*Clostridium perfringens* (CVCC2027, CVCC2030)	Thymol	MIC: 375 µg/mL; MBC: 750 µg/mL	In vitro broth dilution in MHB	Checkerboard assay; combination of thymol and carvacrol showed additive effect	[221]
	Carvacrol				
*C. perfringens*	Tannic acid	250, 500, 750, 1000 mg/kg diet.	In vivo broiler Necrotic enteritis model	Improvements in anti-inflammatory markers, barrier-associated indicators, and microbiota shifts	[224]
*Listeria monocytogenes*	Resveratrol	MIC: 200 µg/mL	In vitro planktonic MIC + biofilm experiments	Strong biofilm-inhibition even at subinhibitory concentrations	[248]
*L. monocytogenes* ATCC 19115	Thymol + Cinnamaldehyde	125 µg/mL thymol + 125 µg/mL cinnamaldehyde	Food model with transcriptomics	Reduced survival and virulence-associated transcriptional activity on meat	[232]
Avian pathogenic *E. coli* (APEC)	Resveratrol	MIC: 128 µg/mL	In vitro broth dilution, biofilm and motility assays	Biofilm inhibition above 1 µg/mL; structural biofilm effects; biofilm eradication at 32 µg/mL resveratrol + 64 µg/mL florfenicol; highlights synergy with an antibiotic	[238]
Methicillin-resistant *Staphylococcus aureus* (MRSA)	trans-Cinnamaldehyde, Thymol, Carvacrol		In vitro MRSA isolate characterization + compound exposure with virulence gene transcription	Downregulation of key virulence genes; antivirulence plus growth inhibition	[243]
Multidrug-resistant *E. coli*	Matrine	MIC: 6.25 mg/mL	In vitro broth microdilution + synergy assays	Checkerboard synergy assay; matrine doses reduced the effective MIC of berberine hydrochloride markedly when combined	[241]
	Berberine hydrochloride	MIC: 1 mg/mL			
	Matrine + berberine hydrochloride	6.25 mg/mL matrine + 1 mg/mL berberine hydrochloride	In vivo chicken colibacillosis model	Improvements via reduced bacterial load and inflammatory factor modulation	

LB: Luria–Bertani; MBC: minimum bactericidal concentration; MIC: minimum inhibitory concentration; SIC: sub-inhibitory concentration.

## 7. Limitations, Challenges, and Knowledge Gaps

Using PCs in poultry feed has garnered interest due to their potential benefits, including improved growth performance, enhanced immunity, and reduced reliance on antibiotics [249]. However, the practical implementation of PCs is not without challenges. One major limitation is the inconsistency in quality and potency, which can arise from variations in plant sources, geographical location, harvesting times, and extraction methods, leading to unpredictable outcomes in poultry health and performance [250]. Furthermore, PCs have limited shelf life, as exposure to light, heat, or oxygen can affect their efficacy, necessitating careful storage and handling [251]. Poor water solubility and absorption, leading to reduced bioavailability, limit their therapeutic potential [252]. Most of the PCs, being immiscible in water, separate when added to liquid growth media, which complicates experimental setup and therapeutic effectiveness [253]. Determining the correct dosage also presents a challenge; incorrect dosing may result in toxicity or insufficient therapeutic effects, and negative interactions with other feed components or medications are possible [254].

Another important but overlooked limitation is safety. PCs should not be considered intrinsically risk-free simply because they are plant-derived. Some PC preparations have been reported to be safe at defined inclusion levels in poultry feed; however, safety depends on the plant source, extraction method, and concentration of active compounds within the formulation. For example, carvacrol-rich essential oil preparations derived from different plant sources have different recommended inclusion levels in poultry diets. Origanum oil extracted from *Thymbra capitata* (Spanish-type origanum oil; 72% carvacrol) has been recommended at 15 mg/kg feed [255], whereas oregano oil preparations containing 78% carvacrol have been reported at inclusion levels of 22 mg/kg for broilers and 33 mg/kg for laying hens [256]. Therefore, future research should focus more on chemically characterized and standardized active compounds and formulations to improve reproducibility, safety assessment, and dose optimization.

Safety data in target animals in the long term are also limited as many of the available studies are short-term and conducted in controlled experimental conditions rather than following extended commercial use. Additionally, there is limited in vivo safety data on purified active PCs. Curcumin has been frequently mentioned as a beneficial additive; however, high dietary inclusion (2.5–10%) of turmeric as a meal has been associated with hepatic histopathological changes in broilers [257]. In another example, continuous oregano supplementation altered hepatic transcriptomic pathways in broilers, suggesting the potential for chronic exposure to alter hepatic metabolic signaling even in the absence of overt clinical toxicity [258]. Also, residue behavior is compound-specific and not universally insignificant. In broilers supplemented with thyme oil, thymol was detected in plasma, liver, kidney, and muscle tissues, with greater accumulation generally reported in kidneys and relatively low concentrations in edible muscle tissue [259]. A separate study similarly showed that dietary thyme increased thymol concentrations in gut contents and plasma, whereas liver and muscle levels remained very low and close to the limit of quantification [260]. Overdose may also compromise performance prior to apparent toxicity: dietary carvacrol has been reported to decrease feed intake and weight gain in broilers [261], while another study describes non-linear dose–response relationships where high inclusion levels may generate negligible or even negative productive responses [190]. Long-term tolerance studies, liver and kidney function tests, histopathology, reproductive and organ-function endpoints, tissue and egg residue depletion, withdrawal periods, and interaction studies with other feed ingredients or medications should therefore be more routinely incorporated into future studies before broad commercial recommendations are made.

Economic viability is another concern, as the extraction and purification processes for high-quality PCs can be costly, limiting their widespread use in large-scale poultry operations. Palatability issues can occur due to the potent odors or flavors of certain PCs, which may decrease feed intake [17]. However, enhancing feed palatability is not implemented in poultry, as these birds exhibit insensitivity to odor [262]. These limitations highlight the need for further research and careful consideration when integrating PCs into poultry feed strategies.

Direct evidence comparing PCs with conventional antibiotics in poultry is limited and formulation-specific, but available data indicate that some standardized phytogenic products can be biologically competitive with AGPs, particularly for feed efficiency and gut-health traits. A 39-day randomized broiler trial compared a phytogenic feed additive, Digestarom^®^ Poultry (150 mg/kg), to bacitracin methylene disalicylate (500 mg/kg) and found the phytogenic arm had a lower overall FCR (1.860 vs. 1.931), slightly higher cumulative body-weight gain (2018.2 vs. 1969.5 g), higher jejunal villus height, lower jejunal crypt depth, lower cecal coliform counts, and higher *Lactobacillus* counts with no significant difference in mortality [263]. But other available studies either lacked an antibiotic comparator or only reported production and gut-histology endpoints. The economic evidence is even more limited, but one broiler trial found that a 0.5% phytobiotic program had a higher benefit–cost ratio and lower cost per kilogram live weight than an antibiotic-fed group [264]. Hence, PCs should not be marketed as cheaper alternatives to antibiotics but rather should be considered as feed additives that work in some instances and not in others. This depends on the quality of the formulation and the maintenance of feed efficiency, and the consideration of AMR externalities in addition to farm-gate performance.

## 8. Future Perspectives

PC use in poultry production should evolve beyond the simple goal of replacing AGPs and instead focus on resistance-sparing precision nutrition that targets bacterial virulence and limits the transmission of AMR. Economically, the burden of poultry-origin AMR is much larger than animal health and includes production losses, increased veterinary costs, foodborne disease burden, trade restrictions and downstream healthcare costs associated with resistant zoonotic infections. Recent macroeconomic modeling by the World Organization for Animal Health (WOAH) and the World Bank estimated that the cumulative loss in global gross domestic product (GDP) due to AMR in food-producing animals could be in $575 billion between 2025 and 2050 [265]. The poultry sector is one of the most impacted livestock industries due to declines in chicken meat productivity and output. In the U.S. alone, foodborne illness from *Salmonella* and *Campylobacter*, both strongly associated with poultry products, cost more than $28 billion in 2023, in health care costs and lost productivity [266]. These results suggest that the economic impacts of AMR are not only confined to farm-level performance but also extend to the food production and public health systems. Conversely, it is estimated that interventions that can reduce AMR by around 30% can lead to an increase in global GDP by $120 billion [265]. Therefore, investigating PCs as environmentally sustainable alternatives which could potentially enhance production efficiency while reducing the need for antibiotics could benefit the economy. Achieving this transition will require deeper investigation of purified active compounds derived from PC extracts and essential oils, along with rigorous mechanistic validation and improved delivery systems that protect bioactive compounds during processing and administration, enabling the development of stable and commercially viable formulations.

Equally important is the integration of resistome-level monitoring into intervention studies. A large chicken gut resistome survey involving 629 samples identified clinically important genes such as *mcr*, *bla*_NDM_, and *tetX*, with ARG abundance closely associated with MGEs [66]. These findings highlight the need for PC trials to measure not only pathogen reduction but also ARG abundance and mobility indicators. Intervention strategies should also consider production-stage specificity. Differences in resistome composition across the broiler lifecycle [267] suggest that dosing strategies, through drinking water, or via feed, may be optimized for early-life microbiome programming or finisher-phase pathogen suppression. HGT dynamics should be evaluated in vivo. Experimental evidence demonstrates that poultry *E. coli* strains can co-transfer plasmids carrying *mcr-1* and *bla_CTX-M_* under selective pressure [268,269], emphasizing the importance of measuring plasmid persistence, conjugation rates, and fitness costs during PC exposure.

Advances in delivery systems may further facilitate translational application. For instance, microencapsulated essential oil–organic acid blends have been shown to reduce *Salmonella* colonization in the ceca while modulating intestinal barrier function and microbiota composition [270], and thymol nanoemulsions illustrate the potential of nanoparticle-enabled stabilization and targeted release [271,272]. Looking ahead, integrating Hi-C metagenomics with long-read sequencing offers a powerful approach for linking mobile ARGs to their bacterial hosts and plasmids, reconstructing gene transfer networks, and defining host range [273,274,275]. Such approaches will enable routine surveillance of high-risk resistance determinants, including *mcr*, *bla_CTX-M_*, and *bla_NDM_*, and support evidence-based risk assessment for AMR in poultry production systems.

## 9. Conclusions

The global shift toward a post-antibiotic era in poultry production, spurred by regulatory requirements in 2026 and changing consumer tastes, has made PCs essential. This review has assessed their capacity to modulate the interconnected resistome, microbiome, and metabolome dynamics, offering a sustainable substitute for conventional growth promoters. Research on *Salmonella*, *Campylobacter*, and *C. perfringens* shows that PCs like carvacrol, thymol, and cinnamaldehyde lower virulence and break up biofilms at lower concentrations that do not kill bacteria. This means that they can effectively disarm pathogens without the selective pressure of synthetic antibiotics.

Some of these compounds also work as prebiotics, promoting beneficial bacteria like *Lactobacillus* and lowering the number of MGEs that spread resistance. This holistic approach makes the intestinal barrier stronger and stops MDR pathogens from spreading in the environment. But there are problems with translation that make widespread implementation of these compounds hard. For commercial reliability, PCs need standardization strategies like rigorous evaluation of stability, bioavailability and safety along with deeper mechanistic and toxicological characterization. Also, advanced delivery systems like microencapsulation and targeted-release granules are needed to keep volatile bioactives safe from the high temperatures used in feed pelleting and make sure they get to the lower gut.

Combining these bioactives with new digital technologies is the key to the future of sustainable poultry farming. AI-powered formulation platforms can make stage-specific blends better and use multi-omics biomarkers to guess how a whole flock will react. In short, no single alternative can completely replace antibiotics. However, synergistic PC blends backed by precision nutrition and biosecurity are a good option for the industry. To safeguard global food safety, preserve the efficacy of the human antibiotic arsenal, and limit the spread of multidrug-resistant zoonotic pathogens, it is essential to explore promising antibiotic alternatives such as PCs.

## Data Availability

No new data were created or analyzed in this study. Data sharing is not applicable to this article.

## Abbreviations

The following abbreviations are used in this manuscript:

AMR	antimicrobial resistance
AGPs	antibiotic growth promoters
ARGs	antimicrobial resistance genes
AHLs	acyl-homoserine lactones
AI	autoinducers
AI-2	autoinducer-2
APEC	avian pathogenic *Escherichia coli*
CFU	colony-forming units
EPS	extracellular polymeric substances
ESBL	extended-spectrum beta-lactamase
EU	European Union
FDA	Food and Drug Administration
FCR	feed conversion ratio
FICI	fractional inhibitory concentration index
GDP	Gross Domestic Product
GM	gut microbiota
GRAS	generally recognized as safe
HGT	horizontal gene transfer
IHME	Institute for Health Metrics and Evaluation
IgA	immunoglobulin A
LA-MRSA	livestock-associated methicillin-resistant *Staphylococcus aureus*
LMICs	low- and middle-income countries
MBC	minimum bactericidal concentration
MDR	multidrug-resistant
MDROs	multidrug-resistant organisms
MGEs	mobile genetic elements
MIC	minimum inhibitory concentration
MPN	most probable number
MRSA	methicillin-resistant *Staphylococcus aureus*
NARMS	National Antimicrobial Resistance Monitoring System
PCs	phytochemicals
QS	quorum sensing
QSIs	quorum sensing inhibitors
SNPs	single nucleotide polymorphisms
STEC	Shiga toxin-producing *Escherichia coli*
UTIs	urinary tract infections
VFD	Veterinary Feed Directive
VRE	vancomycin-resistant enterococci
WEF	World Economic Forum
WGS	whole genome sequencing
WHO	World Health Organization
WOAH	World Organization for Animal Health
